# Hybrids of Pd Nanoparticles and Metal–Organic Frameworks for Enhanced Magnetism

**DOI:** 10.1021/acs.jpclett.1c01108

**Published:** 2021-05-13

**Authors:** Suhwan Kim, Raeesh Muhammad, Peter Schuetzenduebe, Suresh Babu Kalidindi, Gisela Schütz, Hyunchul Oh, Kwanghyo Son

**Affiliations:** † Department of Energy Engineering, Gyeongsang National University, Jinju 52725, Republic of Korea; ‡ Max Planck Institute for Intelligent Systems, Stuttgart D-70569, Germany; § Inorganic and Analytical Chemistry Department, School of Chemistry, Andhra University, Visakhapatnam 530003, India; ∥ Future Convergence Technology Research Institute, Jinju 52725, Republic of Korea

## Abstract

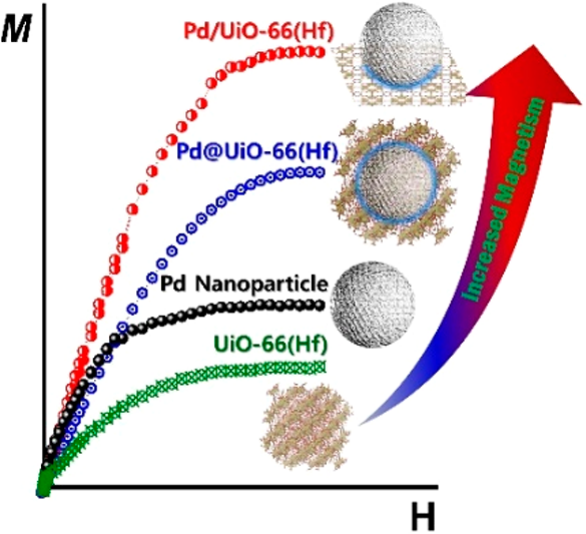

Nonmagnetic Pd exhibits ferromagnetism in the nanosize regime. Various stabilization agents, including surfactants, metal oxide supports, polymers, and porous materials (e.g., metal–organic frameworks (MOFs)), have been employed to prevent the agglomeration of metal nanoparticles. However, magnetic properties are greatly affected by the structural and electronic changes imposed by these stabilizing agents. In particular, metal–MOF hybrids (NPs@MOFs) have reduced magnetic properties, as reported by several authors. Herein, we report the enhancement in magnetic properties resulting from the combination of magnetic Pd NPs with UiO-66(Hf), which exhibits ferromagnetism, and the corresponding modifications in the hybridized structures. These hybridized structures are found to be strongly ferromagnetic, showing high magnetization and coercivity. We observed that the magnetic property is enhanced by 2 to 3 times upon including the Pd NPs on the surface of a UiO-66(Hf) shell support. For a fundamental understanding, the magnetization (*M*–*H* data) of the hybridized structure is analyzed with a modified Langevin function.

Metal nanoparticles (NPs) have attracted significant interest from researchers working in the fields of catalysis, sensors, separations in the biomedical industry, and academia, owing to their exceptional intrinsic properties (i.e., optical properties, electrochemical properties, and magnetism).^1−5^ In particular, NPs have distinct functional attributes, such as high surface-to-volume ratios and number of active sites, that lead to improved performance, as compared to that of bulk materials.^6−8^ For instance, nonmagnetic materials exhibit magnetic behavior by changing their bulk state to the NP form, which is driven by a decrease in metal coordination, changes in local symmetry, and the localization of electrons due to lattice expansion at the surface.^9^ Pd is a representative example of this phenomenon, exhibiting nonmagnetic properties in the bulk phase and ferromagnetic behavior in NPs.^10−15^ To observe the magnetic behavior, the nanometer size has to be preserved. Unfortunately, such NP formation can be induced by a high surface energy, which generally leads to drawbacks, such as agglomeration. Thus, steric hindrance of these metal NPs has been applied with ligands, including thiol, PVP, polymers, or a mixture of different ligands, to prevent NP aggregation and stabilize the metal NPs.^12,16−18^ Alternatively, crystalline porous materials (e.g., metal–organic frameworks (MOFs), which possess a large surface area and a uniform pore structure) have recently been used as encapsulation materials to stabilize NPs through confinement.^19−23^ Owing to their crystalline nature and uniform pore structure, MOFs offer distinctive advantages over conventional porous materials. The pore size and pore–wall functionality of MOFs can also be tuned using pre- and postsynthesis modification methods. Moreover, the feasibility of metal center exchange in MOFs enables changing the magnetic behavior by localizing the spin center. This also makes it possible to explore MOFs as molecular magnets.^24−26^ Thus, a combination of MOFs, as molecular magnets, with encapsulated metal NPs (the hybrid system in metal NPs encapsulated by MOFs) may positively affect the magnetic property.^27^ Despite such hypotheses, only a few studies have been reported on the hybridization of metal NPs and MOFs, and the reports reveal that the magnetism is reduced in most cases. For instance, Zhou et al. recently reported well-defined nanohybrids consisting of a polydopamine-coated NP (FeO) core and an MOF (ZIF-8/UiO-66) shell. Owing to the nonmagnetic properties of the polydopamine coating and MOF components, this core–shell nanohybrid showed a decreased magnetization saturation value.^28^ Elsaidi et al. also reported that the magnetic susceptibility of magnetite (Fe_3_O_4_) microspheres decreased by ∼72% after the growth of an MOF (MIL-101-SO_3_) shell on the magnetite core due to partial oxidation.^27^ Chen et al. similarly reported that Fe_3_O_4_@MIL-100 (Fe) exhibited a lower saturation magnetization value than Fe_3_O_4_ nanospheres.^29^

Herein, we report the observed unexpected magnetic property enhancement produced by a combination of noble metal (Pd) NPs, having surface ferromagnetism, with a core coated by MOFs with weak ferromagnetism. For the encapsulation of Pd NPs, UiO-66(Hf) was used as a single-ion magnet, which was created by an increased NP contact interface. Notably, the encapsulation material (UiO-66(Hf)) should not have any intrinsic magnetic sources because Hf^4+^ and O^2–^ are themselves nonmagnetic ions and the d and f shells of Hf^4+^ are empty and full, respectively. However, the inorganic compound Hf-oxide contains both Hf^4+^ and O^2–^, causing ferromagnetism even at room temperature due to the coupling of oxygen vacancies through exchange interactions or by the mediation of conduction electrons.^30−33^ Such behavior is called d^0^ ferromagnetism and has been observed in powders and thin films;^34^ however, d^0^ ferromagnetism from oxygen vacancies in metal clusters of MOFs has not been investigated thoroughly. Moreover, to the best of our knowledge, no reported studies have investigated the effects of metal NPs on the d^0^ ferromagnetism in MOFs, so we have explored both the ferromagnetism of Pd NPs, arising from the size effect, and the d^0^ ferromagnetism of UiO-66(Hf). For these investigations, we prepared hybridized Pd NP and UiO-66(Hf) composites with two different structures: support and core–shell (Figure 1a)). These two types of hybrid composites may increase the contact interface and interactions of NPs with the encapsulation material (MOF) differently, as Pd NPs are placed on the outer or inner surface of UiO-66(Hf). The details of the synthesis scheme, as shown in Figure 1a, have been reported previously.^35^ The microscopy results indicate that Pd NPs are confined inside the MOFs (Pd@UiO-66(Hf)) or attached on the outer surface of the MOFs (Pd/UiO-66(Hf)), and the average particle size of both systems is *ca*. 7.6 nm.^35^ The Pd contents measured by ICP-OES (EDS) are 4.7 ± 0.4 (7.62) and 5.2 ± 0.3 (4.52) wt % for Pd/UiO-66(Hf) and Pd@UiO-66(Hf), respectively (Figure S1). Although a slight deviation exists between the ICP-OES and SEM-EDS results due to the measurement principles, it implies that the total number of Pd NPs in both hybrid systems is almost same (as obtained via ICP-OES), while the presence of Pd NPs, attached to the outer surface of UiO-66(Hf), is more abundant in Pd/UiO-66(Hf) than in Pd@UiO-66(Hf) (as obtained via SEM-EDS). Consequently, nearly identical loading of 5 wt % Pd NPs with different deposited positions may alter the interparticle distance. The XRD pattern of UiO-66(Hf) matches well with the simulated XRD pattern of UiO-66(Hf). Because the Pd content in both hybridized systems is low (*ca*. 5 wt %), the corresponding peak intensity is also very weak and shows peak broadening. Moreover, the peak position of Pd is overlapped by that of UiO-66(Hf), which is difficult to discern (Figure S2). The Pd 3d XPS curves for the hybridization samples and Pd NPs are found to be asymmetric, and their Pd 3d_3/2_ and 3d_5/2_ peaks are deconvoluted into two curves for Pd^0^ (red lines, binding energy of 334.38 eV) and Pd^2+^ (green lines, binding energy of 336 eV), as shown in Figure 1b.^36,37^ This shows that oxidation occurred in the Pd NPs of all samples and that Pd/UiO-66(Hf) is least oxidized compared to the others. The influence of oxidation in the Pd and UiO-66(Hf) hybrid system can also be seen in the Hf 4f XPS curves, albeit weakly (Figure 1c)), in which two sets of doublets for the Hf 4f_7/2_ and 4f_5/2_ peaks, Hf^4+^ (red lines) and Hf^*x*+^ (black lines) are deconvoluted.^38^ The pure Hf metal peaks are invisible because Hf 4f has higher oxophilicity.^39,40^ The Hf^*x*+^ peaks at 17.13 eV are observed in all of the samples, whereas the peaks of Hf^4+^, which can be a magnetic source under oxygen vacancies, are shifted in the spectra. The binding energy values and fractions of Pd^0^/Pd^2+^ and Hf^4+^/Hf^*x*+^ are collated in Table 1.

**Figure 1:**
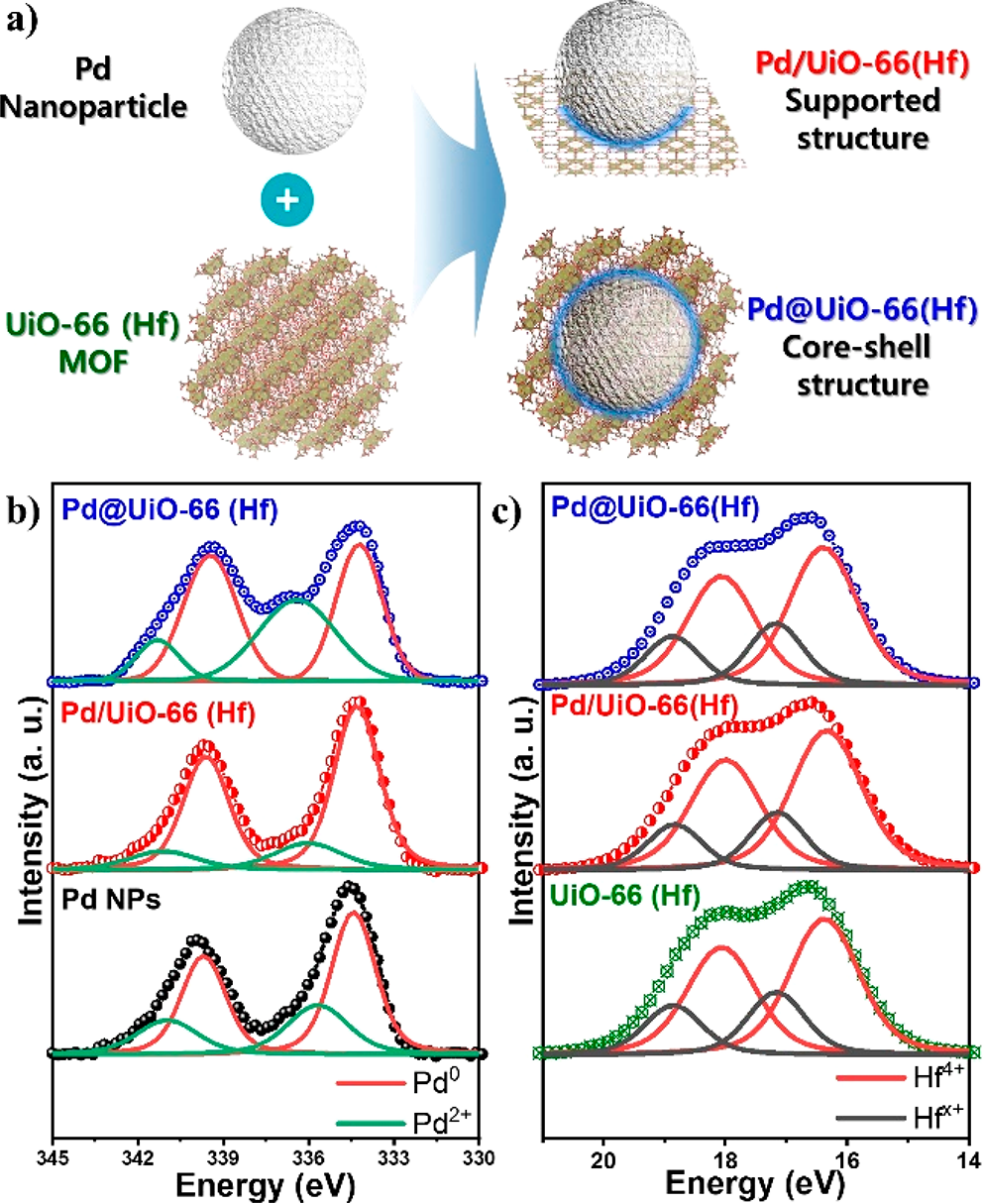
Schematic structure and XPS spectra. (a) Synthesis scheme of the Pd/UIO-66(Hf) support and Pd@UiO-66(Hf) core–shell structure materials. (b) Pd 3d and (c) Hf 4f XPS spectra of Pd NPs, UiO-66(Hf), and the hybridization samples.

**Table 1:** Summary of Magnetic Properties (*M*_S_, *H*_C_, *M*_0pd_, and μ_pdeff_) at 3 K and XPS Spectra (Binding Energy Values and Pd^0^/Pd^2+^ and Hf^4+^/Hf^*x*+^ Fractions for All Samples)

					Pd 3d_5/2_			Hf 4f_7/2_		
	*M*_S_ (emu/g)	*H*_C_ (Oe)	*M*_0pd_ (emu/g)	μ_pdeff_ (μ_B_)	Pd^0^ (eV)	Pd^2+^ (eV)	Pd^0^/Pd^2+^	Hf^4+^ (eV)	Hf^*x*+^ (eV)	Hf^4+^/Hf*^*x*+^*
Pd@UiO-66(Hf)	0.065	10	0.451	1.62	334.17	336.42	51/49	16.43	17.13	74/26
Pd/UiO-66(Hf)	0.090	210	0.487	3.46	334.38	336.02	81/19	16.29	17.13	76/24
UiO-66(Hf)	0.025							16.43	17.13	72/28
Pd NPs	0.037	52	0.041	6.26	334.38	335.61	68/32			

The magnetic properties of all of the samples were compared, and the ferromagnetic behaviors were observed to be enhanced as a result of the combination of Pd NPs and MOFs. As mentioned earlier, the surface ferromagnetism of Pd NPs and the defect magnetism of Hf^4+^ in UiO-66(Hf) are verified in Figures 2 and 3. The ZFC-FC magnetic susceptibilities are shown in Figure 2a as functions of temperature up to room temperature at 0.05 T. The χ–*T* curves include the diamagnetic signal of ligands, and the remaining residual paramagnetic signals emerge from within the Pd NPs. The susceptibility at low fields shows a linear relation between the magnetization and the external field in the para- and diamagnetic materials. These results show that Pd NPs and Pd/UiO-66(HF) exhibit strong paramagnetic effects, whereas UiO-66(Hf) and Pd@UiO-66(Hf) show weak paramagnetism. The magnetic susceptibilities change according to the strength of the external magnetic field (Figure S4-1); for instance, the order of magnitude of the susceptibility changes at 0.5 T. Accordingly, we observe a reversal in the susceptibilities (i.e., hybridized sample susceptibilities become higher than those of the pristine samples (Figure S4-2)). Furthermore, bifurcations, which are evidence of ferromagnetic properties, are found in all samples. The *M*_FC_ – *M*_ZFC_ and d(*M*_FC_ – *M*_ZFC_)/d*T* analyses yield information on the magnetic phase transitions and interparticle interactions.^41^ Pd NPs show ferromagnetic behavior at all temperature ranges, as expected.^42^ In the case of UiO-66(Hf), ferromagnetism was confirmed, although it was not very strong. The magnetic susceptibility of ligands in UiO-66(Hf) was calculated to be −41 × 10^–6^ emu/g by using Pascal’s constant (χ_D_). (Details are given in SI S4.^43^) The negative susceptibility of the ligands was found to be small in the experimental raw data. The positive value of susceptibility can be attributed to the presence of d^0^ ferromagnetism and paramagnetic behavior from the Hf ion. The crossing point of the ZFC and FC curves in UiO-66(Hf) can be observed at approximately 270 K, above which the paramagnetic behavior is dominant. The existence of d^0^ ferromagnetism in HfO_2_ thin films has been reported by several authors.^32,44,45^ In the case of UiO-66(Hf), the source of magnetism can also be Hf^4+^ with oxygen vacancies. However, ferromagnetic behavior in UiO-66(Hf) has been observed only at low temperatures. In the case of Pd@UiO-66(Hf), the surface magnetism of Pd NPs was reduced because its contact interface was larger than that of Pd/UiO-66(Hf). The increased oxidation at both sites reduced the magnetic properties overall. The difference in the number of Hf ions and oxygen vacancies in UiO-66 (Hf) and the hybridized samples is about ±2%. The Pd/UiO-66(Hf) support structure shows a stronger susceptibility due to the preservation of pure Pd composition. In particular, Pd/UiO-66(Hf) exhibits different behavior during the ZFC and FC measurements (Figure 2a and Figure S4 under various magnetic field conditions). The *M*_FC_–*M*_ZFC_ curve of Pd/UiO-66(Hf) is obtained as a sum fitted to the material fraction of Pd NPs and UiO-66(Hf) (Figure 2b). The d(*M*_FC_ – *M*_ZFC_)/d*T* curve shows two peaks (one positive and another negative (inset in Figure 2b)) which can be ascribed to the changes in interaction among the Pd NPs or between the Pd NPs and the MOF.^41^ The interaction between the Pd NPs and MOF is weak during the ZFC measurement; however, it is strong during FC measurements.

**Figure 2:**
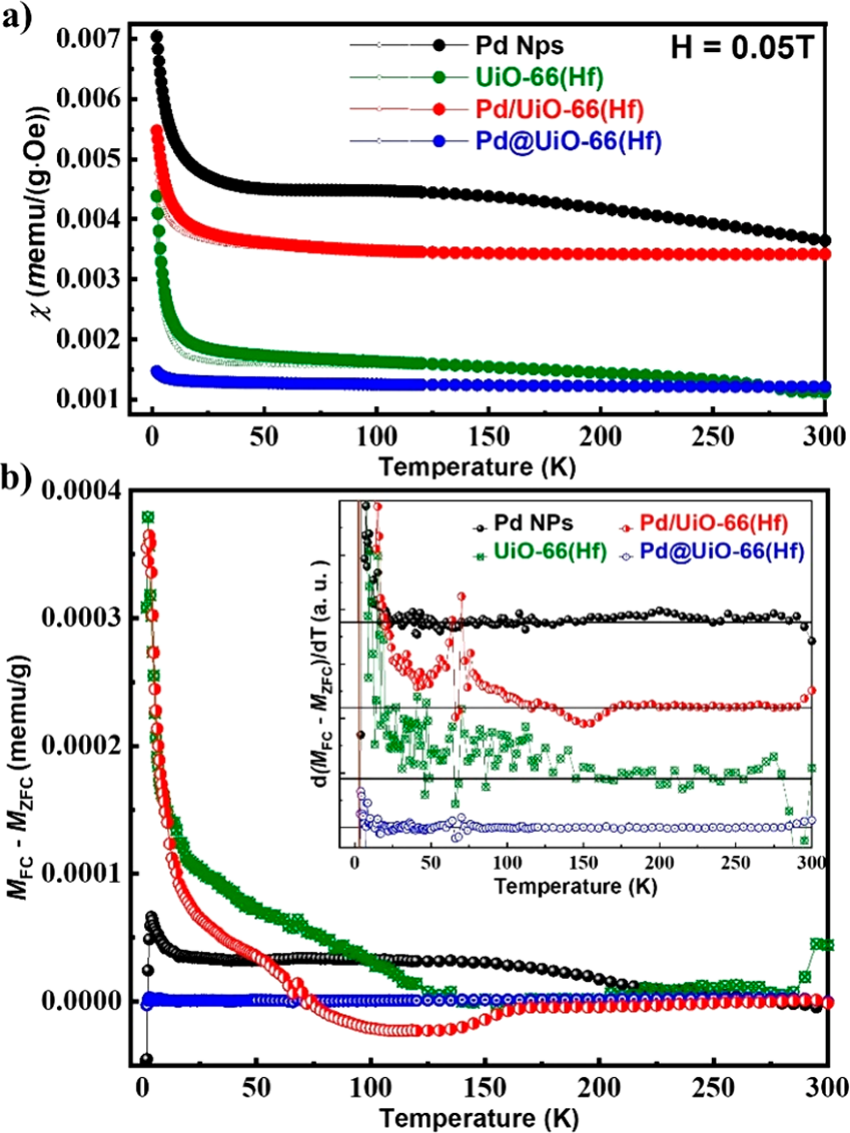
Temperature-dependent magnetic properties. (a) Temperature-dependent magnetic susceptibility at 0.05 T and (b) *M*_Fc_ – *M*_ZFC_ curves (inset: derivatives) for Pd NPs (black), Pd/UiO-66(Hf) (red), Pd@UiO-66(Hf) (blue), and UiO-66(Hf) (green).

**Figure 3:**
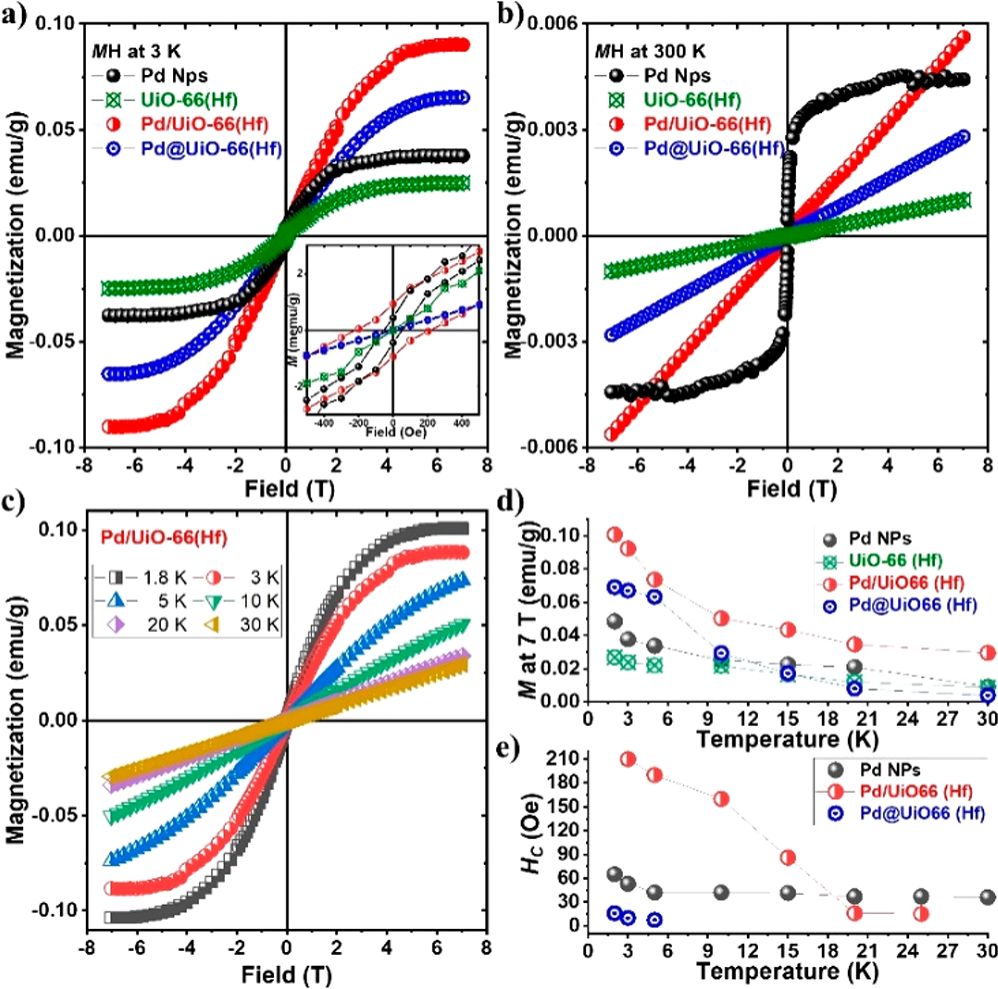
Field-dependent magnetic property. (a) Corrected M–H curves at 3 K. (Inset) Enlarged view of the M–H curves around 0 Oe. (b) Corrected M–H curves at 300 K for Pd NPs, Pd/UiO-66(Hf), Pd@UiO-66(Hf), and UiO-66(Hf). (c) Hysteresis loop of magnetization for Pd/UiO-66(Hf) at 1.8, 3, 5, 10, 20, and 30 K. (d) Magnetization (*M* at 7 T) and (e) coercivity (*H*_C_) of Pd NPs, Pd/UiO-66(Hf), Pd@UiO-66(Hf), and UiO-66(Hf).

The structure-dependent variation in the magnetic behavior of the studied samples can be observed in their *M*–*H* curves. Figure 3a displays the field-dependent magnetization curves, at 3 K, which shows the corrected linear diamagnetic and paramagnetic data. The hysteresis loops of the samples clearly indicate their ferromagnetic behavior at low temperatures. The samples with Pd NPs exhibit coercivity with high magnetization. The *M*_S_ and *H*_C_ values at 3 K are summarized in Table 1. As further supporting data, raw *M*–*H* curves without the correction are shown in Figure S5. The hybridization with MOFs increases the saturation magnetization to twice its original value in both samples, in contrast to the Pd NPs without MOF. However, the enhanced ferromagnetic parameters disappear at high temperatures (Figure 3b), where only paramagnetic slopes are observed. Interestingly, the Pd/UiO-66(Hf) still shows a higher susceptibility at higher temperature. Figure 3c shows the hysteresis loops of Pd/UiO-66(Hf) at various temperatures. The temperature-dependent magnetization and coercivity are shown in Figure 3d,e. The magnetic properties of the hybridized samples are enhanced at low temperatures. Beyond 15 K, the ferromagnetic phenomena, such as magnetization and coercivity, decrease abruptly, and the magnetic signal of Pd@UiO-66(Hf) reduces to that of pristine UiO-66(Hf). The Pd NPs in the Pd@UiO-66(Hf) core–shell structure are almost in full contact with UiO-66(Hf), which leads to a higher PdO ratio in the Pd NPs, thereby increasing the interactions on all surfaces. The surface effect on Pd NPs in Pd@UiO-66(Hf) vanishes with increasing temperature. Thus, the ferromagnetic features (higher magnetization and small coercivity) are observed only at low temperatures. However, in the case of Pd/UiO-66(Hf), the partial surface contact provides an advantage to both Pd and UiO-66(Hf), which causes a reduction in the oxidation of Pd NPs and the interaction between particles. This reduced interaction leads to a higher magnetization and coercivity at higher temperatures.^46^ Details on the ferromagnetism of pristine samples (Pd NPs and UiO-66 (Hf)) are explained in SI S5.

The *M*–*H* curves at various temperatures were analyzed using a modified Langevin function that represents the superparamagnetic behavior of ferromagnetic NPs. The raw *M*–*H* data, excluding the paramagnetic and diamagnetic components, exhibit nonsaturating behavior with increasing field strength; therefore, they cannot be analyzed by a simple Langevin fitting. Consequently, we used a modified Langevin fitting function with temperature-dependent susceptibility (eq 1), expressed as^47,48^
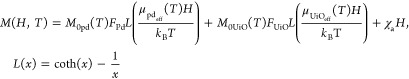
1where *M*(*H*, *T*) is the measured magnetization; *F*_Pd_ (*F*_UiO_ = 1 – *F*_Pd_) is the mass fraction of the Pd NPs in the sample; χ_a_(*T*) is the susceptibility of the Pd core, ligands, etc.; and *M*_0pd_(*T*) (*M*_0UiO_(*T*)) and μ_Pdeff_(*T*) (μ_UiOeff_(*T*)) represent the magnetization (emu/g) and the effective magnetic moment (μ_B_), which is the average magnetic moment of the uncompensated for spins in the particles, respectively. The *M*–*H* curves, from 0 to 7 T, at 1.8, 3, 5, and 10 K were selected and analyzed. Above the selected temperatures, the ferromagnetic characteristics have vanished, and only the approximate paramagnetic characteristics have remained both temperature- and field-dependent magnetic properties in hybrids samples. Figure 4a,b show the fitting results for Pd/UiO-66(Hf) and Pd@UiO-66(Hf), respectively. The temperature dependence of the obtained fitting parameters is shown in Figure 4c,d. We can see that the obtained magnetization (*M*_0_(*T*)) values are enhanced in both Pd and UiO-66(Hf) due to the hybridization. Although the magnetization of individual Pd NPs is large, the number of Pd NPs is approximately 5% in both samples. Therefore, the actual total magnetic moment follows the UiO-66(Hf) magnetization trend. The structure-dependent interactions can be ascertained from the values of the obtained effective moments, μ_Pdeff_ and μ_UiOeff_. In the support sample, both Pd and UiO have their respective effective moments; therefore, μ_Pdeff_ ≈ constant may be attributed to the uncompensated for moments. On the contrary, in the core–shell structure, the effective moments of both Pd and UiO increase together and have similar values; as a result, μ_eff_ also increases with the increasing temperature, which can be attributed to the intraparticle interactions in the magnetic cores of Pd and UiO particles. As expected with magnetic properties, Pd/UiO-66(Hf) has higher magnetization and effective moment than Pd@UiO-66 (Hf) due to a larger ferromagnetic region in the Pd nanoparticles.

**Figure 4:**
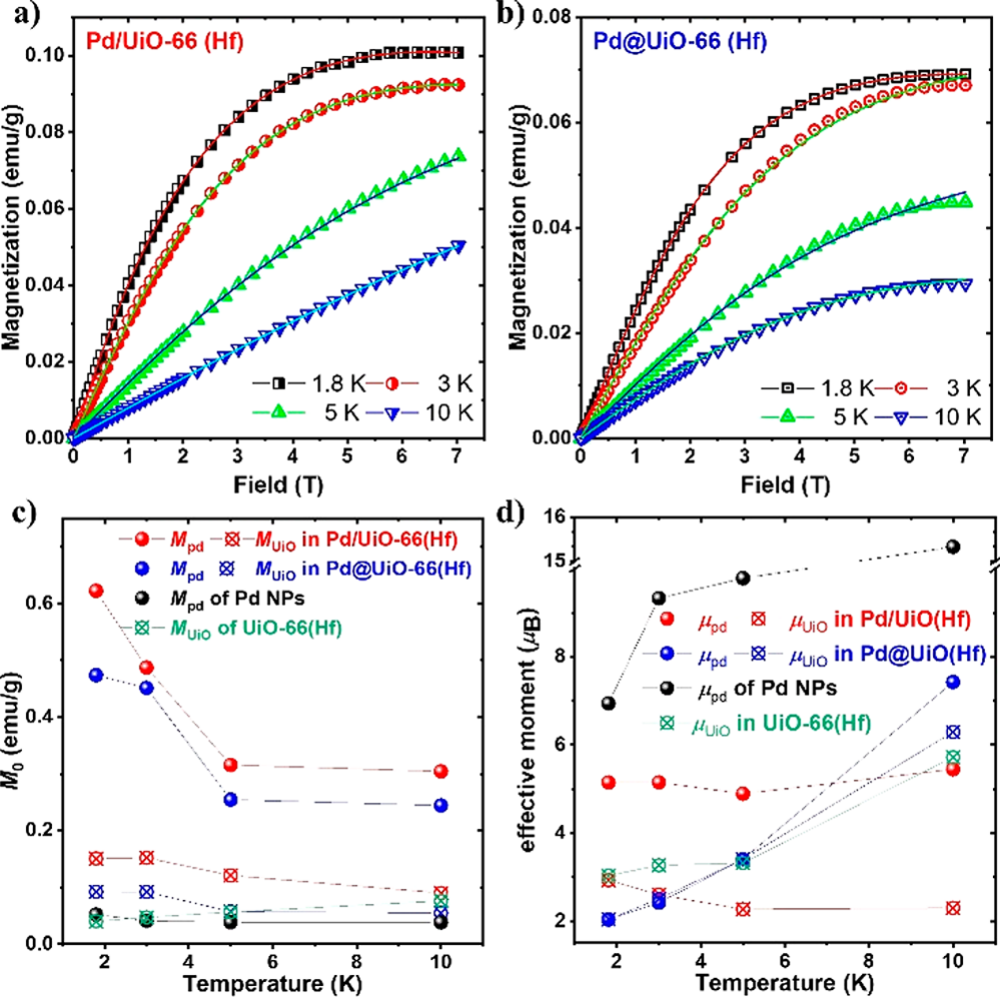
*M*–*H* curve analysis with magnetization as a function of the applied field at the indicated temperatures. Solid lines show the fitting results for (a) Pd/UiO-66(Hf) and (b) Pd@UiO-66(Hf). (c) Temperature dependencies of the fitting parameters, *M*_Pd_ and *M*_UiO_, and (d) μ_Pdeff_ and μ_UiOeff_.

In summary, we conducted a detailed study of the magnetic properties of Pd NPs, UiO-66(Hf) MOF, and hybridized material systems via magnetic property measurements and analysis with a modified Langevin function. We confirm the existence of surface magnetism in noble metals and d^0^ ferromagnetism in UiO-66(Hf). The magnetism of Pd NPs is due to the surface effect, and the magnetism of UiO-66(Hf) arises from the existence of oxygen vacancy defects. As the two materials are hybridized, the surface effect of Pd NPs is maintained by the preserved particle sizes, resulting in further enhanced ferromagnetic behavior. In particular, in the supported structure, the Pd NPs become attached to the outer surface of the MOF, which provides the NPs with a free and oxidized Pd surface. By contrast, the core–shell structure induces oxidation at the interface because Pd NPs are well-distributed inside the MOFs. Consequently, the intraparticle interactions and interparticle interactions between the NPs and MOFs, which are associated with magnetic properties, can also be affected depending on the structure. From these results, we can conclude that the hybridization of noble metal NPs and MOFs strengthens the magnetism of the hybrid structure, which occurs as a result of the position of the NPs, contact interface, oxygen states, and interactions between the NPs and MOFs. The contact interface plays a significant role in the modulation of magnetic properties. The combination of Pd and Hf prevents the oxidation of Pd NPs, which is advantageous because Hf, with its high oxophilicity and inherent magnetism, is more suitable than other materials for producing magnetization in metal NPs. Additionally, this report provides an important and efficient approach to verifying the enhancement of ferromagnetism in hybridized materials and highlights the differences in magnetic properties resulting from structural changes in such hybridized materials.

## Experimental Methods

*Synthesis of Hybrid Pd Nanoparticles and Metal–Organic Frameworks*. The Pd/UiO-66(Hf) support sample was synthesized by stirring UiO-66(Hf) in colloidal Pd NPs. The Pd@UiO-66(Hf) core–shell sample was prepared by using the “bottle around a ship” approach via the solvothermal condensation of UiO-66(Hf) precursors, HfCl_4_·6H_2_O and 1,4-benzenedicarboxylate, in DMF containing presynthesized Pd NPs.^35^

*Characterization*. The hybridization structures of Pd NPs, UiO-66(Hf), and hybridized structure samples were characterized by transmission electron microscopy (TEM) imaging carried out using JEOL 2100F, an FEG source, a 200 kV source voltage. The crystal structures were verified via powder X-ray diffraction (PXRD) using Cu Kα radiation (Rigaku Ultima IV). The elemental composition and impurity were analyzed using inductively coupled plasma optical emission (ICP-OES), scanning electron microscopy with energy-dispersive spectroscopy (SEM-EDS), and X-ray photoemission spectroscopy (XPS). The magnetic properties of the Pd-MOF hybrid systems were investigated via a commercial superconducting quantum interference magnetometer (SQUID, Quantum Design MPMS3). Temperature-dependent magnetization was measured in the temperature range of 2–300 K with various external magnetic fields ranging from 0.01 to 2 T by using the zero-field-cool (ZFC) and field-cool (FC) protocols. Field-dependent magnetization was measured by varying the magnetic field (*H*) over the range of −7 T ≤ *H* ≤ 7 T, where a 7 T external field was sufficient to saturate the magnetization. The diamagnetic component of the capsule and ligands in the MOFs and the paramagnetic component inside Pd NPs were removed from measured *M*–*H* values with the subtraction of a linear fit at high fields. Furthermore, nonmagnetic tools were used to perform the measurements, and extra precautions were taken to prevent contamination of the magnetic metals.

## Supporting Information Available

Experiment results of XRD, TEM, EDS, XPS, magnetic properties (measurements), and Pascal’s constant calculation modified Langevin fittings (PDF)
