# Supplementary material for: Hybrids of Pd Nanoparticles and Metal–Organic Frameworks for Enhanced Magnetism
Source: J Phys Chem Lett. 2021 May 13;12(19):4742–8. doi: 10.1021/acs.jpclett.1c01108 (PMC8279731; doi:10.1021/acs.jpclett.1c01108)
Supplement: Supplementary file 1 — jz1c01108_si_001.pdf [file jz1c01108_si_001.pdf]

Supplementary Information

Hybrids of Pd Nanoparticles and Metal–Organic Frameworks for Enhanced Magnetism

Suhwan Kim,[†] *Raeesh Muhammad,*[†] *Peter Schuetzenduebe,*[§] *Suresh Babu Kalidindi,*[♀]

Gisela Schütz,[§] *Hyunchul Oh*,*[†, ǁ] *and Kwanghyo Son*,*[§]

[†]Department of Energy Engineering, Gyeongsang National University, Jinju 52725, Republic of Korea

[§]Max Planck Institute for Intelligent Systems, Stuttgart, D-70569, Germany

[♀] Inorganic and Analytical Chemistry Department, School of Chemistry, Andhra University, Visakhapatnam 530003, India

[ǁ]Future Convergence Technology Research Institute, Jinju 52725, Republic of Korea.

S1. Energy dispersion X-ray Scan (EDS) results of the hybridization samples

S2. The crystal and morphology structures

- -X-ray Diffraction (XRD) pattern
- -Transmission electron microscopy (TEM)

S3. X-ray Photoemission Spectra (XPS) Survey and Oxygen energy

S4. Temperature dependent magnetic properties with various external fields

- -ZFCFC of samples at various field
- -M_{FC}-M_{ZFC} at 0.5 T analysis.

S5. MH curves of samples with raw Pd NPs result

S6. The ferromagnetism of pristine samples (Pd NPs, and UiO-66(Hf))

S7. Raw data of MH curves for Pd/UiO-66 (Hf) and Pd@UiO-66 (Hf)

S8. Temperature dependence of *M*s and *H*c

S9. Langevin fitting results of Pd nanoparticles

S1. Energy dispersion X-ray Scan (EDS) results of the hybridization samples

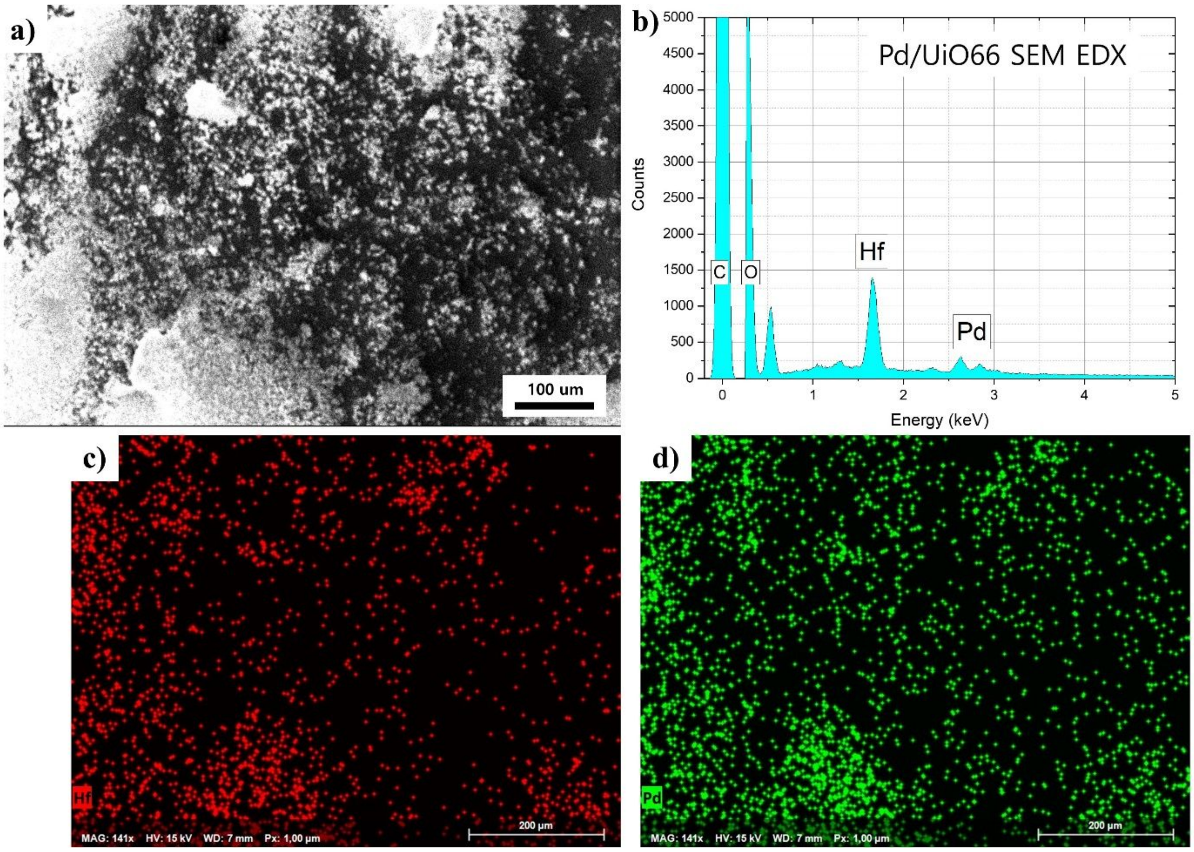

Figure S1-1. SEM image, EDS spectrum and elemental mapping images of Pd/UiO-66(Hf) (mapping color Hf (red) and Pd (green))

Element	At. NO.	Mass [%]	Mass Norm. [%]	Atom [%]
Hf	72	51.64	92.38	88.22
Pd	46	4.11	7.62	11.78
	**Sum**	55.75	100	100

Table S1-1. Mass and atom ratio of Hf and Pd in Pd/UiO-66(Hf) from EDS spectrum

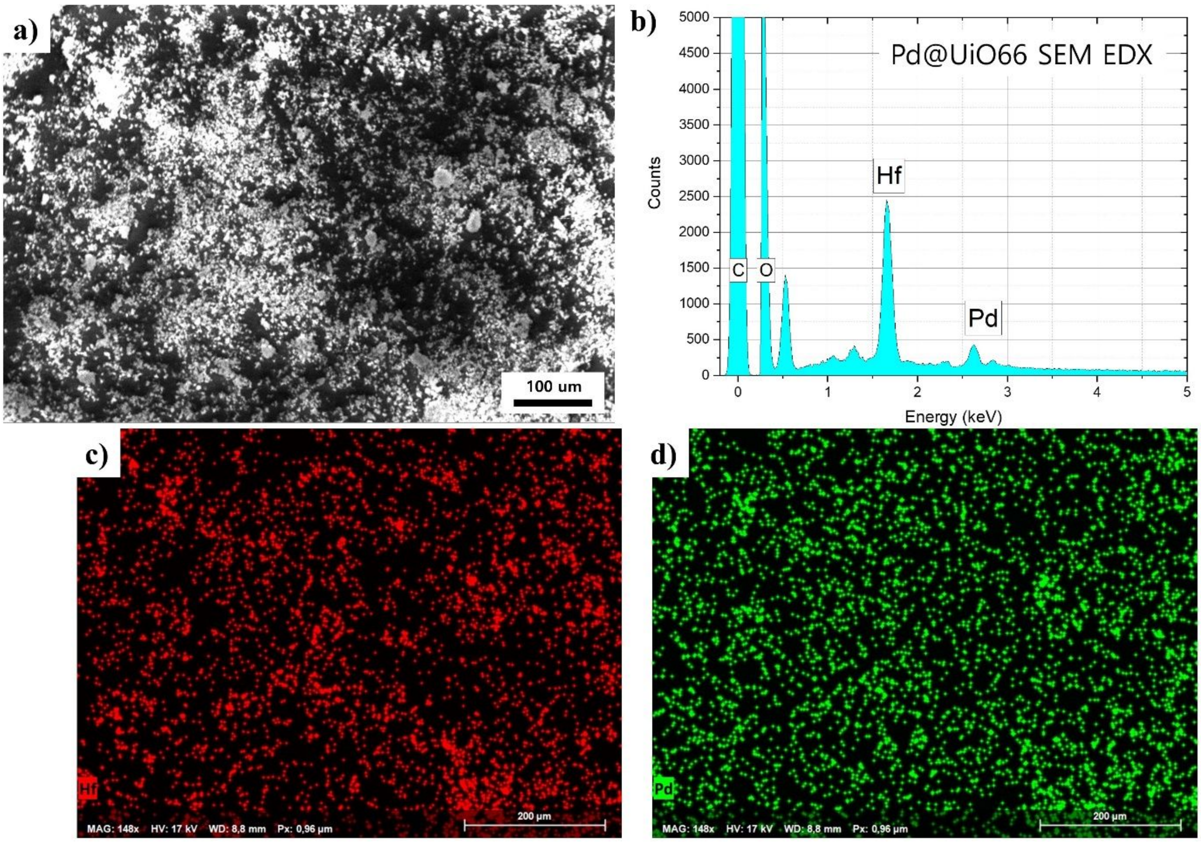

Figure S1-2. SEM image, EDS spectrum and elemental mapping images of Pd@UiO-66(Hf) (mapping color Hf (red) and Pd (green))

Element	At. NO.	Mass [%]	Mass Norm. [%]	Atom [%]
Hf	72	57.01	95.48	92.48
Pd	46	2.76	4.52	7.52
	**Sum**	59.77	100	100

Table S1-2. Mass and atom ratio of Hf and Pd in Pd@UiO-66(Hf) from EDS spectrum

The EDS results that detected Pd atoms in Pd/UiO-66(Hf) are more than Pd@UiO-66(Hf) represent that the Pd NPs are on the surface in Pd/UiO-66(Hf). The actual amount of Pd can be more accurately known from the ICP-OES and BET results presented in previous studies. The Pd contents are is 4.7 ± 0.4 wt.% for Pd/UiO-66(Hf) and 5.2 ± 0.3 wt.% for Pd@UiO-66(Hf).

The BET results that are 895 $m^2g^{-1}$for UiO-66(Hf) pristine, 801 $m^2g^{-1}$for Pd/UiO-66(Hf), and 791 $m^2g^{-1}$for Pd@UiO-66(Hf) indicate the presence of Pd NPs in UiO-66(Hf). [4-5]

S2. The crystal and morphology structures

-X-ray Diffraction (XRD) pattern in the Pd structure region

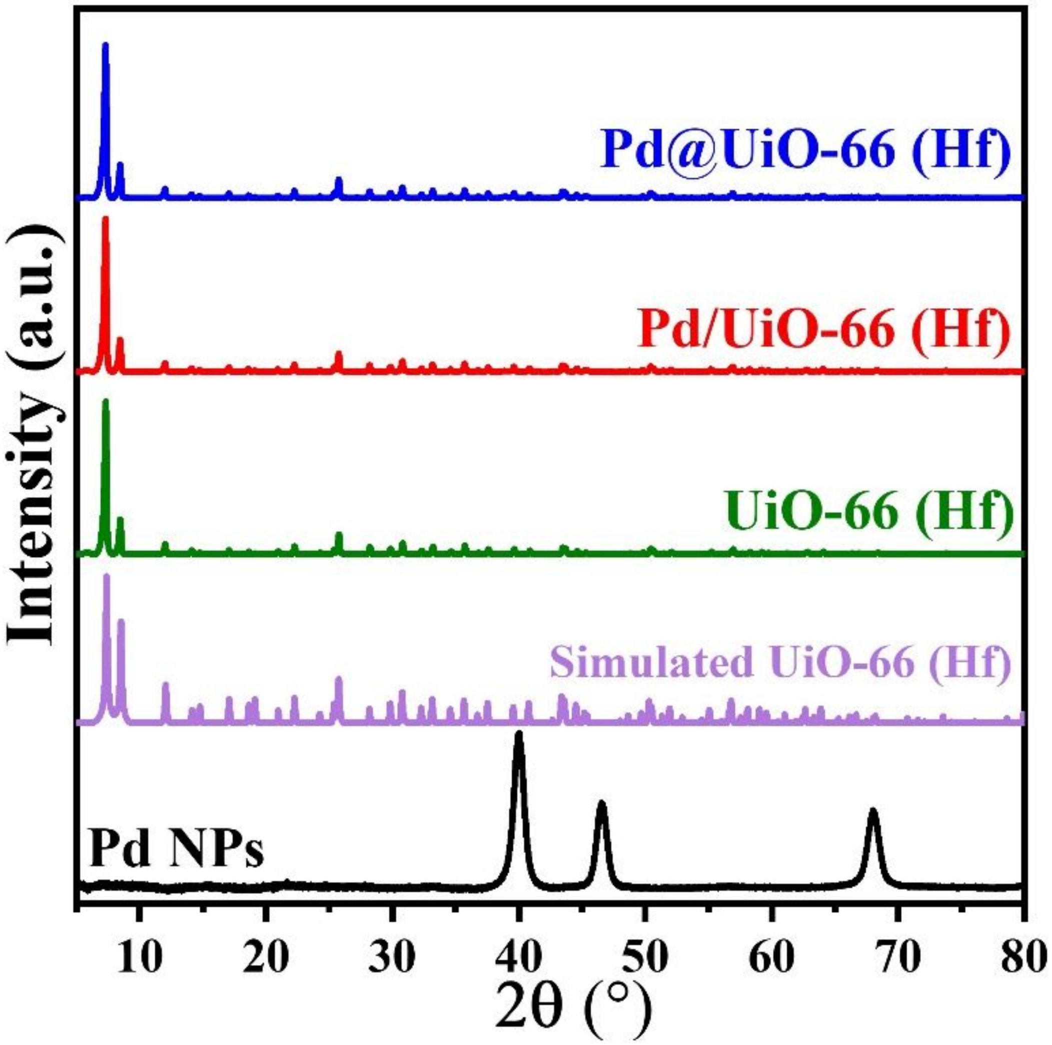

Figure S2-1. XRD patterns of Pd NPs, UiO- 66(Hf), simulated UiO-66(Hf), Pd/UiO-66(Hf) support, and Pd@UiO-66(Hf) core-shell structure materials.

-Transmission electron microscopy (TEM)

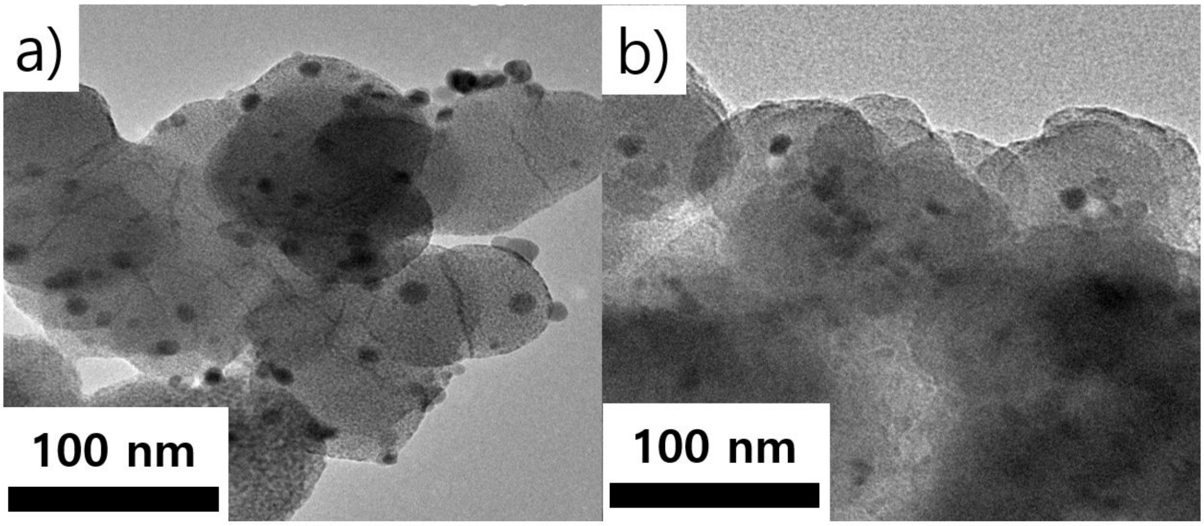

Figure S2-2. TEM images. Bright field images a) for Pd/UiO-66(Hf), b) for Pd@UiO-66(Hf).

S3. X-ray Photoemmission Spectra (XPS) Survay and Oxygen energy

XPS measurements were carried out in a Thermo VG Thetaprobe system (Thermo Fisher Scientific, USA) employing monochromatic Al Kα radiation (*hv*=1486.68 eV) produced with an electrical power of 100 W. The X-ray spot size on the sample was about 400μm in diameter. A flood gun was employed to compensate possible surface charging. No futher peak position correction was done. The base pressure of the used XPS was 8x10^{-10} mbar. The samples are loaded into the UHV one day before all three samples are measured. Survey spectra were recorded with a pass energy of 200 eV and more detailed spectra of single elemental peaks were measured in snapscan mode afterwards. The measurements were fitted using the fitting routines included in the XPS software Avantage.

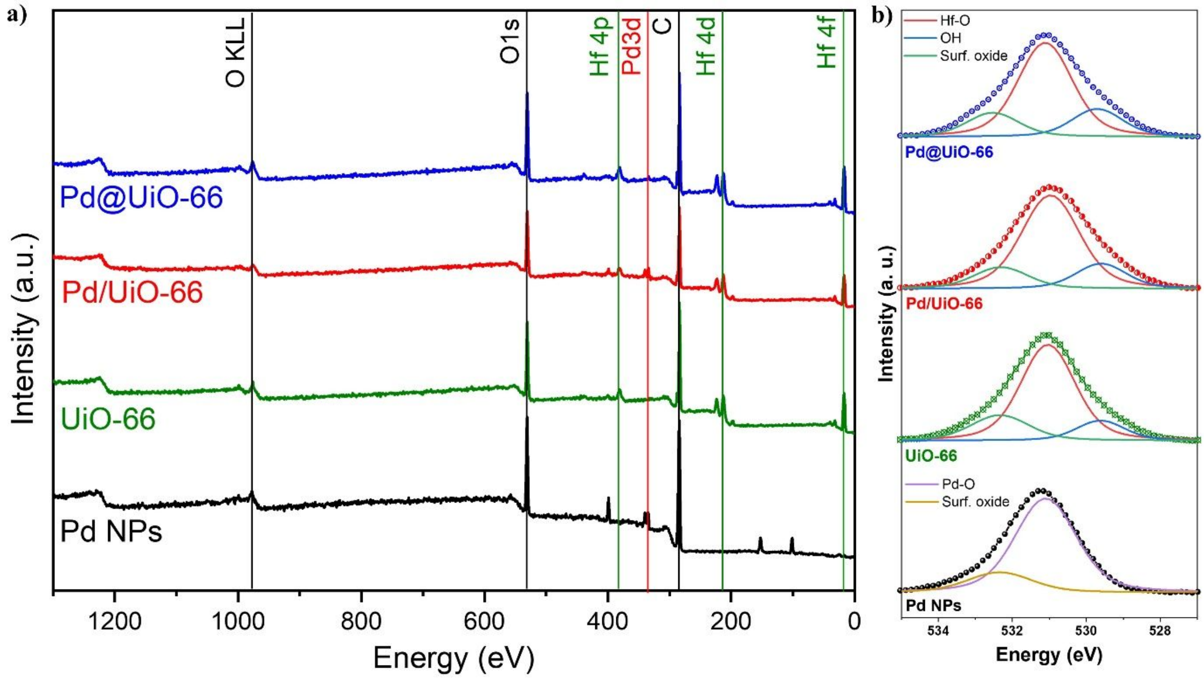

Figure S3. XPS spectra. a) Survey spectra and b) O 1s XPS spectra of all sample Pd NPs(black), UiO-66 (green), Pd/UiO-66 (red), and Pd@UiO-66 (blue).

	O 1s				O 1s		
	OH (eV)	Hf(Pd)-O (eV)	Oxygen vacancy (eV)	OH/Hf(Pd)-O/vacancy	Pd-O (eV)	Surf. Oxide (eV)	Pd-O/Surf. Oxide
Pd@UiO-66(Hf)	529.666	531.157	532.498	18/66/16			
Pd/UiO-66(Hf)	529.666	531.008	532.349	16/69/15			
UiO-66(Hf)	529.666	531.008	532.349	13/68/19			
Pd NPs					531.157	532.349	82/19

Table S3 Summary of binding energy values and oxygen fraction of all samples.

The OH spectrum is from ligands. The oxygen vacancies exist on the surface of UiO-66(Hf). The oxygen ratios are also changed due to the hybridization structure. Pd/UiO-66(Hf) shows higher Hf(Pd)-O and lower oxygen vacancy because Pd NPs are located on the surface of UiO-66(Hf). In Pd@UiO-66(Hf), oxygen on the surface is maintained and the reason for reducing the Hf(Pd)-O ratio is because Pd and Hf share oxide at the interface.

S4. Temperature dependent magnetic properties with various fields

-ZFCFC of samples at various field

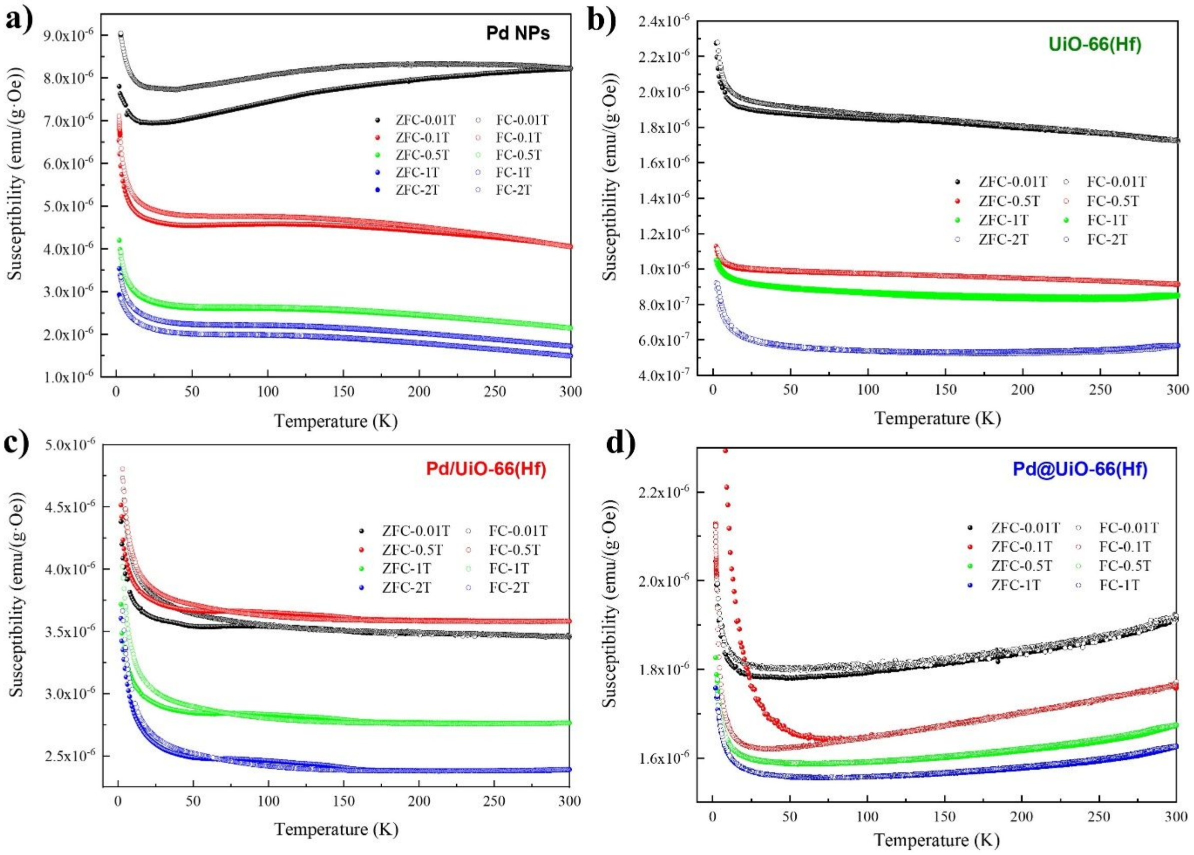

Figure S4-1. Susceptibilities as functions of temperature for a) Pd NPs, b) UiO-66(Hf), c) Pd/UiO-66(Hf) and d) Pd@UiO-66(Hf) measured after zero-field cooling (ZFC) and field-cooling (FC) at various magnetic-field strengths.

In Fig. S4c), the shape difference between ZFC and FC of Pd/UiO-66(Hf) was confirmed at all external magnetic fields.

The Pascal's constant calculation.

The Pascal's constant value of UiO-66(Hf) was estimated by the following process.

χ_D (UiO-66 without Hf) = χ_D ($O_4(OH)_4[(O_2C)$-C_6H_4-$(CO_2)]_6$)

=4 χ_D (O)+4 χ_D (OH)+6[χ_D (C)+ χ_D (O2) + χ_D (Benzene)+ 4χ_D (H)+ χ_D (C) +χ_D (O2)]

=4(-4.6)+4(-12.0)+6[(-6)+(-12)+(-18)+4(-2.93)+(-6)+(-12)]

=-458.72 × 10^{-6} emu/mol = -4.1 × 10^{-7} emu/(g Oe)

In the case of Pd/UiO-66(Hf), Pd@UiO-66(Hf) and UiO-66(Hf), the raw data without capsule signal have the diamagnetism of the ligands remain.

-M_{FC}-M_{ZFC} at 0.5 T analysis.

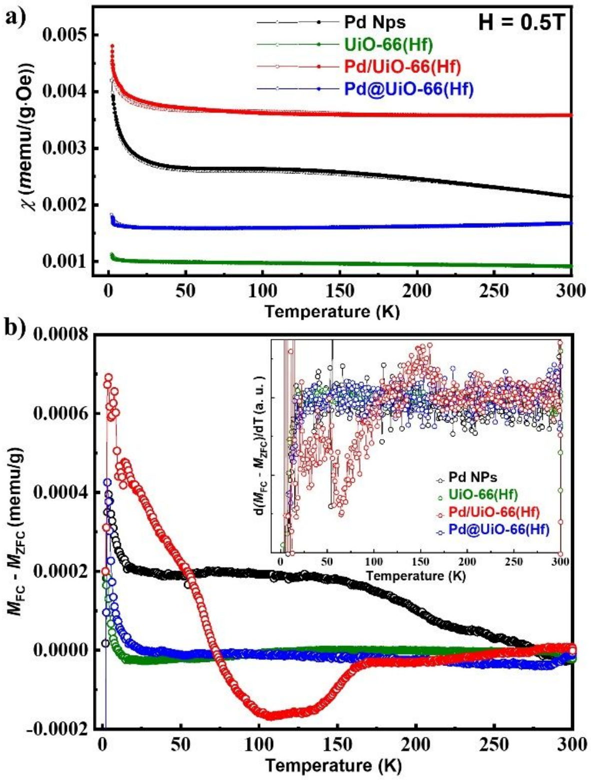

Figure S4-2. Temperature dependent magnetic property. a) Temperature dependent magnetic susceptibility at 0.5 T and b) M_{Fc}-M_{ZFC} curves (inset: derivatives) for Pd NPs, Pd/UiO-66(Hf), Pd@UiO-66(Hf), and UiO-66(Hf).

S5. MH curves of samples with raw Pd NPs result

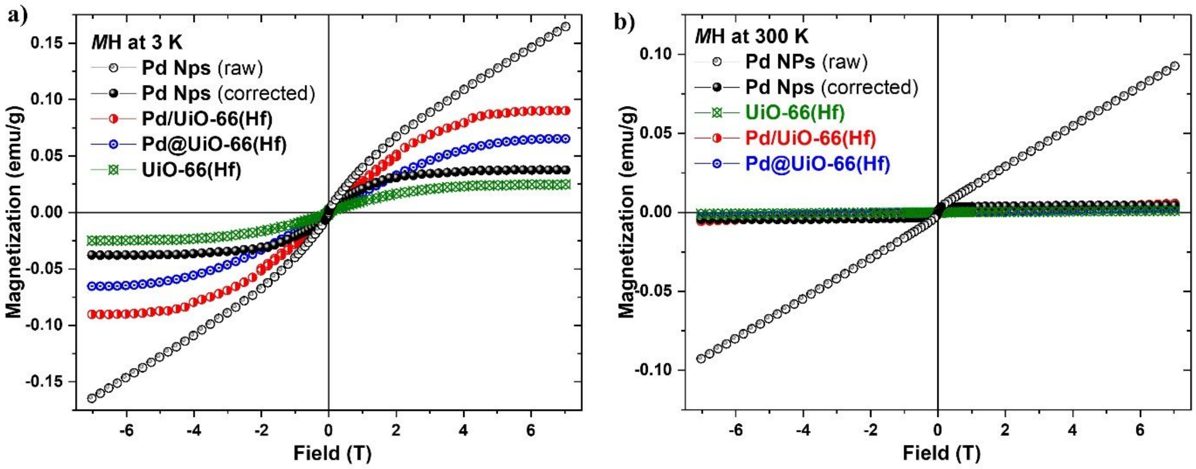

Figure S5. M-H curves with raw data of Pd NPs at a) 3 K and b) 300 K.

The core in Pd NPs has strong paramagnetic behavior. The figure 2 b) and c) show only ferromagnetism on surface effect. Other samples are not shown remarkable change between raw and correction.

S6. The ferromagnetism of pristine samples (Pd NPs, and UiO-66(Hf))

The ferromagnetism of Pd NPs

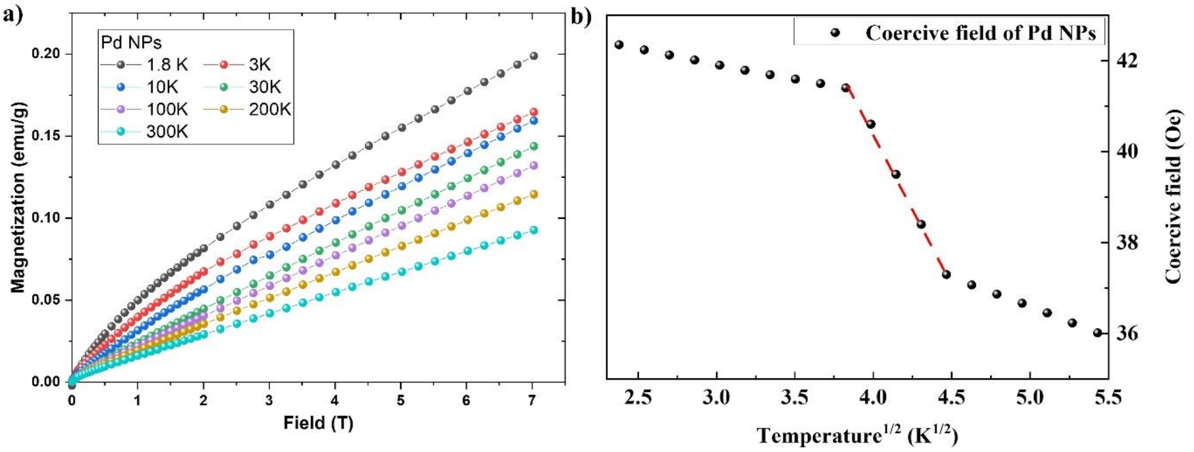

Figure S6-1 a) MH curves at various temperatures. b) Coercivity of Pd NPs as a function of the root square of temperature.

The paramagnetic slop of Pd core is strong in MH curves without paramagnetic correction in all range of temperature. (Figure S6-1 a)) The monodomain behaviour of Pd NPs can be explained by the Stoner–Wohlfarth model, defined mathematically as $H_c(T)=H_c(0)[1-(T/T_B)^{1/2}]$. [1] Because surface magnetisation is large and non-negligible in the high-temperature region, the magnetic field applied to the surface increases the total magnetic field around the core, and the coercive field decreases.

As the temperature decreases, the coercive field increases again because the surface spin becomes saturated as it merges into the core cluster.[2] We were able to obtain a blocking temperature of 280 K using the Stoner–Wohlfarth model fitting (Figure S6-1 b)). The bifurcation of the ZFC and FC curves was confirmed at the 275 K point of the M–T curve, and this value was almost identical to the value obtained with the Stoner- Wohlfarth model. Additionally, the increase in coercivity with decrease in temperature could be due to the anisotropy effect of the magnetic NPs beyond the bulk value.[3]

The ferromagnetism of UiO-66 (Hf)

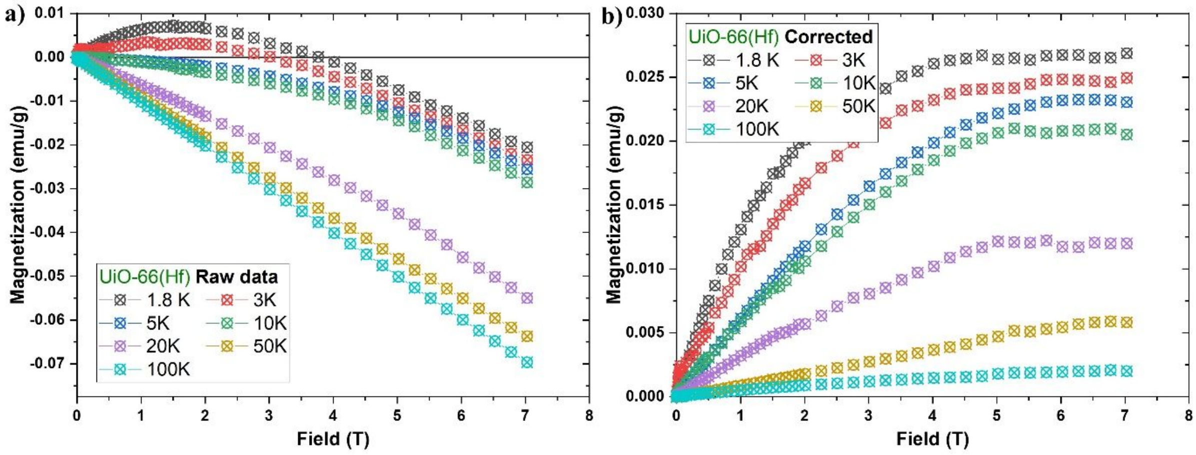

Figure S6-2 MH curves at various temperatures. a) raw data and b) corrected diamagnetic term.

The diamagnetic behavior of ligand is dominant in UiO-66(Hf) (Figure S6-2) The corrected MH curves for UiO-66 (Hf), shown in Figure S6-2 b), confirm the superparamagnetic behavior, and ferromagnetism can be observed at low temperatures. Moreover, the paramagnetic behavior of Hf-O dominated at temperature beyond 20 K.

S7. Raw data of MH curves for Pd/UiO-66 (Hf) and Pd@UiO-66 (Hf)

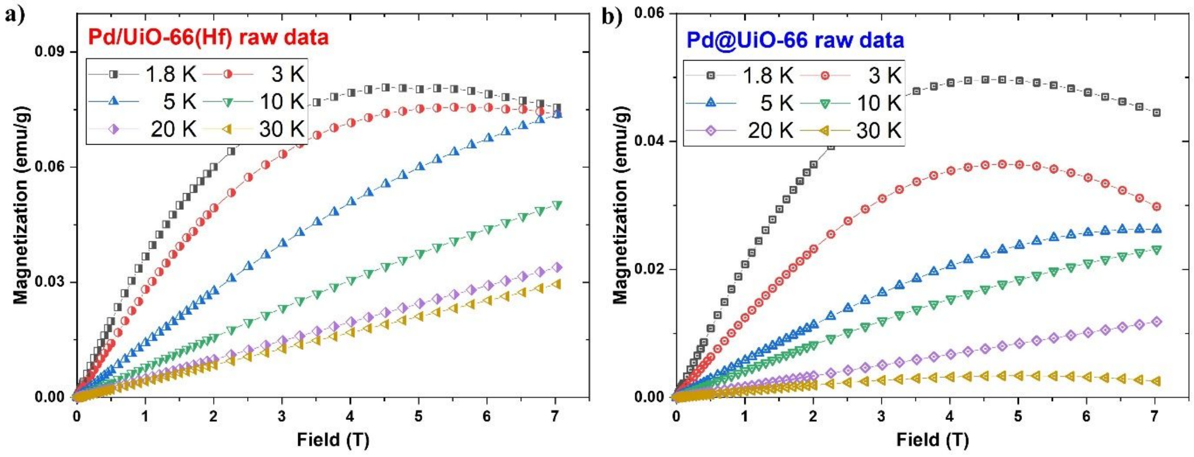

Figure S7. The raw MH curves without correction at various temperatures. a) Pd/UiO-66(Hf) and b) Pd@UiO-66(Hf)

Fig. S7 shows the raw MH curves without correction. The diamagnetic signal would not have affect in the hybridization samples. The small amount of Pd NPs are dominant.

S8. Temperature dependence of *M*s and *H*c

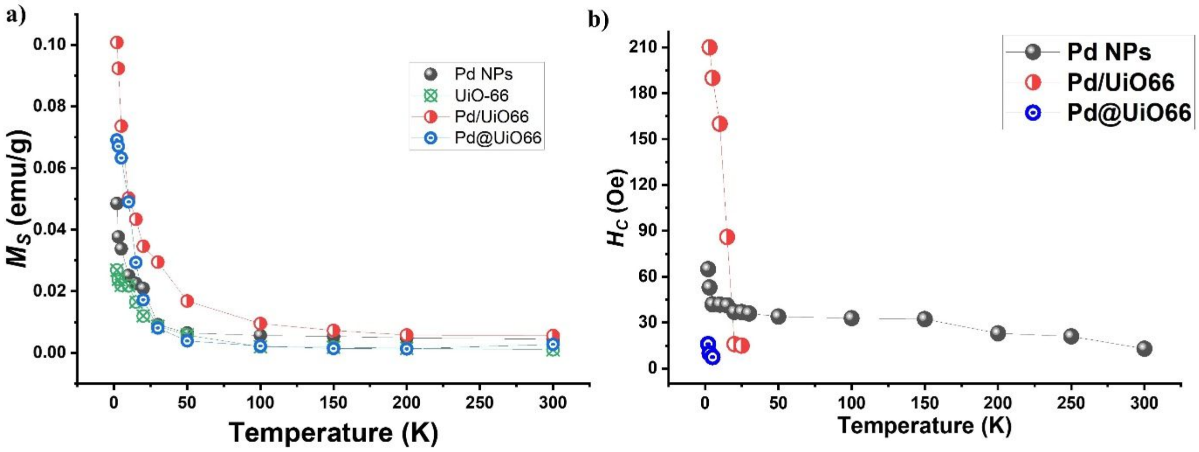

Figure S8. Temperature dependence of a) saturation magnetization and b) coercivity.

Pd NPs show ferromagnetic characterization with high saturation magnetization and coercivity in the full range of temperature

UiO-66(Hf) also has ferromagnetism due to HfO_2. In the raw data, the strong diamagnetic signal from ligands were confirmed (Fig. S6-2).

The Pd/UiO-66(Hf) shows enhanced ferromagnetic properties. The saturation magnetization is the highest till 300 K. Though the coercivity is disappear above 30 K.

The Pd@UiO-66(Hf) is also enhanced magnetic behavior at low temperature. However, it follows UiO-66(Hf) pristine above 20 K.

S9. Langevin fitting results of Pd nanoparticles

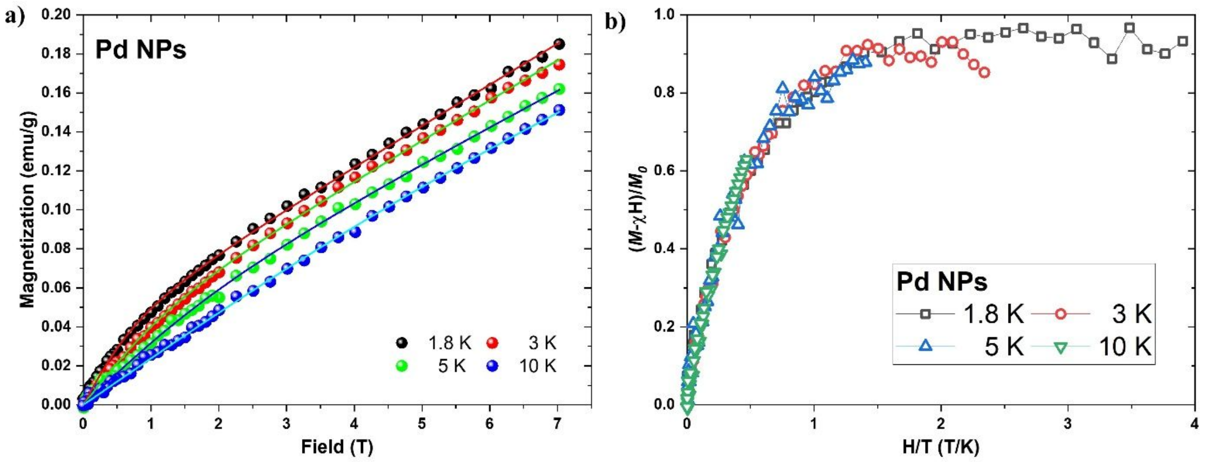

Figure S9. a) MH curves withtou paramagnetic correction. b) universal curve for Pd NPs

MH curves at low temperature for the modified Langevin function fitting are shown in Fig. S9. The particle effective moment is bigger than the hybridization samples, because, the fitting results of hybridization sample are the average of Pd NPs and HfO_2 signals. To check it and compare the signal, S10 shows removed UiO-66(Hf) signal, which is obtained UiO-66(Hf) expereimental data.

References

(1) Stoner, E. C.; Wohlfarth, E. *Philosophical Transactions of the Royal Society of London. Series A, Mathematical and Physical Sciences* **1948,** 240, 599-642.

(2) Qin, W.; Li, X.; Xie, Y.; Zhang, Z. Reentrant paramagnetism induced by drastic reduction of magnetic couplings at surfaces of superparamagnetic nanoparticles. *Phys. Rev. B* **2014,** 90, 224416.

(3) Tripathy, D.; Adeyeye, A.; Boothroyd, C. Effect of Fe_2O_3 on the transport and magnetic properties of half metallic Fe_3O_4. *J. Appl. Phys.* **2006,** 99, 08J105.

(4) Bakuru, V. R.; Velaga, B.; Peela, N. R.; Kalidindi, S. B. Hybridization of Pd Nanoparticles with UiO-66(Hf) Metal-Organic Framework and the Effect of Nanostructure on the Catalytic Properties. *Chem. Eur. J.* **2018,** 24, 15978-15982.

(5) Bakuru, V. R.; Davis, D.; Kalidindi, S. B. Cooperative catalysis at the metal-MOF interface: hydrodeoxygenation of vanillin over Pd nanoparticles covered with a UiO-66(Hf) MOF. *Dalton Trans.* **2019**, 48, 8573.
